# Using virtual reality to understand gun owner decision-making

**DOI:** 10.3389/fpubh.2025.1679347

**Published:** 2025-12-16

**Authors:** Cassandra K. Crifasi, William Wical, Ethan C. Bartlett, Alexander D. McCourt, Silvia Villarreal, Julie A. Ward

**Affiliations:** 1 Department of Health Policy and Management, Center for Gun Violence Solutions, Johns Hopkins Bloomberg School of Public Health, Baltimore, MD, United States; 2 Department of Medicine, Health, and Society, College of Arts and Sciences, Vanderbilt University, Nashville, TN, United States

**Keywords:** firearm, firearm owners, virtual reality, safety training, decision-making

## Abstract

**Background:**

Nearly 70% of gun owners support requiring concealed carry license applicants to pass a test demonstrating safe and lawful weapon use. However, many states are removing training requirements and gun owners’ behaviors and decision-making are under-examined. We sought to assess whether virtual reality (VR) could provide insight into these elements in the context of public carrying.

**Methods:**

We created a novel VR system with a practice-focused firing range and two public-space scenarios involving (1) alleged shoplifting and (2) armed robbery. Participants completed scenarios in random order, then participated in a semi-structured debriefing interview which explored actions and decisions within the scenarios, impressions of system mechanics, and perceived realism. Interviews were audio recorded, professionally transcribed, and inductively coded to identify themes.

**Results:**

Ten gun owners completed the study. In both scenarios, participants reported wanting to avoid weapon use to prevent escalating tensions. In the robbery scenario, intentions to intervene were notably dependent on perceived risk of physical harm to self or others. Although participants described some mechanical distractions related to holster non-visibility and an underweighted mock handgun, all stated their behaviors represented real-life behavioral intentions.

**Conclusion:**

To our knowledge, this is the first immersive VR system to test gun owners’ behaviors and decision-making in public carrying situations. Participants’ gun use was influenced by non-player characters’ actions and the presence of another gun. These findings suggest VR may create meaningful and low-risk environments for teaching or evaluating safe public carrying if firing range or other training access is limited.

## Introduction

Over the past 50 years, there have been substantial changes to the ways states regulate public carrying of concealed guns. In the 1980s, most states did not allow for public concealed carry; among those that did, a permit was required, with robust application criteria including a criminal background check and training requirements (1). Currently, however, more than half of states have removed the permitting requirement—allowing for so called “permitless” carry—such that anyone who can lawfully own a gun is allowed to carry concealed in public without any training or background check requirements (1). Relaxing or removing concealed carry permitting requirements has been associated with increases in crime and violence. For example, some states require training courses with a hands-on, live fire component, but the adoption of permitless carry removes this requirement for carrying in public. Among the 4 states that adopted permitless carry that resulted in the loss of a live fire safety training requirement between 1981 and 2019, this change was associated with a 32% increase in the rate of assaults with guns (2).

This shift toward permitless carry is counter to public opinion on the matter. In a 2023 nationally representative survey of US adults, only 23% supported allowing permitless carry, and 73% supported requiring concealed carry license applicants to pass a test demonstrating they can safely and lawfully use their firearms in common situations they might encounter (3). However, even in states with permitting requirements that include training, few have proficiency requirements, and none require a test of decision-making. The feasibility of implementing such publicly popular assessments may be constrained by zoning, space, and cost, in addition to a lack of legal requirements.

Virtual reality (VR) technology has become an increasingly popular and accessible way to test, educate, and train professionals across various domains such as law enforcement, medicine, education, and industry (4, 5). For example, VR systems are frequently used by law enforcement and the military to support simulated shooting lessons and training for critical incident response. These systems present simulations that emulate real-life scenarios, providing opportunities to evaluate individual performance in terms of appropriate, accurate, and precise weapon use (4). Such VR-facilitated evaluations indicate that standard training often fails to adequately prepare officers to respond to the kind of situations they face while on duty. Highly realistic scenarios in a VR system allow people the opportunity to experience how both psychological and physiological factors affect performance and decision-making (6, 7). Turning to the gun-owning public, this prior work presents an opportunity to learn from the experiences of trained professionals who routinely carry firearms in public and must make complex decisions about when (and when not) to use them.

Carrying a firearm to protect the public in emergency situations comes with serious physical, mental, and psychological demands (8). With the expansion of public concealed carry, everyday citizens may be engaged in high-risk situations where their decision-making with a firearm could impact the life and safety of themselves or others. However, few, if any, VR systems currently exist to assess performance and decision-making among civilian gun owners. To address this gap, we developed and pilot-tested a novel VR system to explore the feasibility of VR for either potential training or assessment of gun owners’ firearm uses in public encounters or both.

## Methods

### VR system

For this project, we created and pilot-tested a VR system that presented situations that gun owners might encounter while carrying a firearm in public. The VR system included a firing range and two scenarios. The firing range was created to support the participants with familiarizing themselves with both the VR system generally and the VR handgun specifically. We consulted with law enforcement and legal experts in the development of the scenarios to identify potential situations individuals carrying firearms in public might encounter when it may or may not be legally and/or situationally appropriate to use their firearm. The two scenarios took place in a simulated convenience store. The first scenario involved a customer non-player character (NPC) accused of shoplifting by the cashier NPC. The second scenario involved a robber NPC armed with a handgun entering the store and demanding cash from the cashier NPC. These were designed to create situations where firearm use by the participant could be deemed legally and situationally inappropriate (shoplifting) or ambiguous if not defensible (armed robbery). The cashier NPC was a young, white woman and the customer/robber NPC was a young man, randomized to be either White or Black presenting. While not considered in this study given the pilot nature to assess feasibility and small sample size, future work may consider whether there are differential responses in the same scenario depending on the race and gender of the customer/robber NPC. Future iterations of the system may also randomize the characteristics of the cashier NPC.

To assess the feasibility of using the system, in this pilot project, participants were randomized to which scenario they experienced first. The only instructions participants received prior to beginning the scenarios were: “you are traveling alone and driving through your home state. You stop at a convenience store to buy a bottle of water. This area is familiar, but you have not been inside this store before. After you begin, walk inside to look for a bottle of water.” These instructions were intended to inform participants they will be in a generally familiar environment (i.e., their home state where they should be familiar with relevant firearm laws) and a generally unfamiliar environment (i.e., a convenience store in which they had not previously been as we could not have recreated a known store for each participant). After entering the store and walking to the refrigerators, the randomized scenario was automatically initiated. Scenarios ended if a participant fired their weapon or when the non-cashier NPC left the store.

### Participant recruitment and experience

Gun owners were recruited for the pilot testing through shooting clubs and gun ranges using a snowball sampling strategy. Data collection occurred between April and July 2024. Interested participants were screened for being a current firearm owner, over the age of 18, and living in Maryland to assure consistent “home state” assumptions. Prior to participating, all individuals completed written informed consent. They also completed a pre-survey that included questions on demographics, experience with firearms, types of firearms owned, history of firearm training, and experience with VR systems.

Ten participants completed the VR pilot. Most were active duty or retired military or retired law enforcement (*n* = 8). All had received formal firearms training, either through their occupation or as part of concealed carry licensing requirements. Few had any experience with immersive VR. As these individuals were highly trained, that could have influenced their behaviors. However, given their high level of training, their perspectives on realism and functionality of the system during the pilot phase could increase our confidence in the use of the system in a more general population. Upon completion of the experience, individual semi-structured debriefing interviews were conducted to explore participants’ thoughts, decisions, and behaviors within each scenario. Interviewers also inquired about the mechanical or technical feasibility of the system and the perceived realism of VR and the specific scenarios. On average, participants spent 15 min in the VR system and 18 min in the debrief interview. Participants received a $100 Visa gift card for their time. Interviews were audio recorded and transcribed verbatim. This project was reviewed and approved by the Johns Hopkins Bloomberg School of Public Health Institutional Review Board.

### Data analysis

The interview transcripts were analyzed using MAXQDA, a qualitative data analysis software. Since there has been relatively little research conducted on using VR to understand how civilian gun owners make decisions on when to use their firearms, we used an inductive and iterative coding approach. All codes were defined and assigned representative quotes to ensure consistent coding throughout the analysis. Interviews were coded using a three-wave coding strategy—open, axial, and focused. After open coding was completed, emergent themes were discussed and agreed upon by research team members. Relationships between codes were discussed, which informed the completion of the axial phase of coding. Lastly, focused coding was completed to determine how specific codes related to broader themes. Only themes that reached saturation were included in this manuscript. As research has shown, qualitative analysis of the narratives of six participants offers a sufficiently robust sample to establish the majority of codes for a specific topic (9). Two primary themes emerged: concerns related to avoiding conflict versus strategic escalation as a primary factor in decision-making and VR as an effective tool for understanding behavior of gun owners.

## Results

At no point did participants unholster their weapon during the shoplifting scenario. While most participants reported not feeling in danger (*n* = 8), two people noted caution around the possibility of the cashier using a weapon to solve the dispute. In contrast, during the robbery scenario, half of participants (*n* = 5) drew and fired their gun at the assailant. The robber was shot by four out of five participants (one person unintentionally missed). Additionally, two people unholstered their gun but did not shoot at the robber, as the assailant left the store with the money.

### Reflections on decisions and behaviors

In both scenarios, participants noted that their decision to draw and fire or leave the gun holstered was primarily driven by two main factors. First, they reported a desire to not use their firearm when it would further escalate the situation—this was particularly true for the shoplifting scenario. Second, participants reported feeling as if the situation was already escalated by the presence of an armed NPC in the robbery scenario. Critically, perceptions of needing to involve themselves and potentially escalate the situation shifted throughout the robbery scenario depending on the perceived threat of bodily harm to themselves or the NPC cashier.

#### Avoiding unnecessary escalation

Participants did not feel as though it was appropriate to draw their firearm during the shoplifting scenario because, as one participant (P5) noted, “Getting involved would just escalate the situation needlessly.” Decision-making around not intervening was largely related to beliefs that if there was no risk for bodily harm, drawing a weapon was an inappropriate response. As one participant (P3) stated:

“I didn't see him with his weapon out, so I didn't think there needed to be a weapon involved at all, because people have disagreements all the time, and I think that that is excessive force. And so, the fact that it was just a disagreement, again, material things are material things that can be replaced, and if there wasn't a threat, there's no real need for it.”

Similarly, another participant (P10) explained that shoplifting incidents should involve the police rather than other civilians and that the only reason to intervene would be a threat to someone’s life. They stated, “Nobody’s life is being threatened in this scenario, so I did not think I had any business to say anything, do anything, get involved in any way, shape, or form.” In the shoplifting scenario, participants reported watching the body language—specifically the hands—of the people arguing to determine if there was going to be an escalation.

#### Responding to escalation by another person

At no point did participants draw their weapon unless they had visually confirmed that there was already a firearm involved in the robbery scenario. In these cases, when someone else had escalated a situation, participants noted the importance of their safety and that of the convenience store clerk. As one participant (P1) stated, “[It’s] the same kind of thought process [in both scenarios] of like ‘Does this concern me? Is anyone in imminent danger?’ Okay, I have something that can resolve this one way or another. Should I?” Similarly, this participant, after firing a shot which resulted in the robber fleeing the scene, did not feel as though it was important to pursue him because the situation did not require any further escalation. Importantly, determinations of when to escalate or not were contingent upon feeling safe. Multiple participants cited their prior experiences with being in the military or police force as important factors in informing their decision-making process on when escalation was appropriate.

### Reflections on realism and technical merits of VR

All participants felt as though VR was an effective tool for testing, educating, and reinforcing skill-oriented behaviors related to firearm usage. One participant (P10) reflected that VR can allow you the flexibility to behave in ways you would not be allowed to in a traditional firing range:

“I thought the virtual environment was great. Again, this is the first time I've done that, but the virtual environment was, I think, a perfect place to learn something like this. Ranges don't typically allow you to just walk around with your gun and potentially unholster it at any time. So I think the VR is a good place to learn this stuff.”

Overall, participants noted three key factors that supported the suitability of this method: comfort with the system, realism of the scenarios, and transferability of decision making.

#### Comfort with system

Nearly all participants reported that they felt that the VR equipment was relatively comfortable and did not meaningfully detract from their participation in the research. The most common criticism participants had was that, while the difficulty of the trigger pull felt real, the gun and holster were not realistic because the firearm was not heavy enough and it was difficult to unholster the gun. As one participant (P5) explained: “The trigger pull increases the realism of it. Would have been nice if there was a rubber wrapper around the handle so it was a little bit bigger.” Another participant (P9) explained:

“I mean, the gun definitely does not feel at all realistic. The extra final trigger, I was like ‘Oh, okay, I have to really squeeze the final…’ That's realistic enough, but in terms of the feel, the weight of the weapon itself, it's not very realistic.”

For some participants, this detracted from the overall immersion of the experience. Participants suggested increasing the width of the handle, weight of the gun, and incorporating pneumatics to provide recoil upon firing would have increased the realism of the virtual experience.

#### Realism of scenarios

All participants reported feeling as if the scenarios were realistic in that they could conceivably happen to them in their everyday lives. However, there were some aspects of the experience that detracted from the overall realism of using VR. One participant (P9) noted that the most significant difference between the VR experience and everyday life was the lack of other things happening in the background that could be a distraction or compete for attention. They noted that this would change their decision making on whether firing their weapon was appropriate, as the safety of bystanders was a primary concern. While the simplicity of the VR experience decreased the realism of the scenarios, this participant still believed the experience was an appropriate means of testing decision making.

Participant perspectives on the realism of the system and the scenarios underscores the potential usefulness of VR for training. For example, one participant (P1) described the benefit of having a reminder that not every situation requires intervention:

“For what I presume to kind of be the goal and intent, which is if you're a gun owner, don't go and look for an excuse to shoot somebody in a convenience store. Think through what is going on. Think through your place. Think how you can do good, and that may be doing nothing, and I think that alone, just doing this little training reaffirms that in my mind, sometimes to do good, do nothing, and that's okay.”

#### Transferability of decision making

Participants overwhelmingly felt as though they would respond similarly in real life as they did in the VR experience. Notably, one participant (P8) stated that while they thought they would likely respond in a similar way in real life, they were unsure because of having never witnessed a shoplifting or armed robbery incident. Participants also indicated that the inability to verbally engage with the NPCs detracted from the transferability of their decision-making to real life. Of the 10 participants, only one described that they would have acted differently in real life; they stated that they would have walked up and tried to diffuse the shoplifting scenario. While the participants could speak to the NPCs, there was a limited set of responses from the VR system. A few participants described this limited their interactions with the system, but not to the point where they felt they would have behaved differently in real life. The most common suggestion on how to best enhance the likelihood of getting representative responses was to increase the ability of participants to interact, communicate with, and potentially change the behaviors of the NPCs.

## Discussion

To the best of our knowledge, this pilot study is the first to examine the feasibility of using VR to explore behaviors among gun owners in the US that can lead to a better understanding of their decision-making process. In our pilot study with 10 generally highly trained gun owners, participants reported that VR was an effective tool to provide real life interactions in a low-stakes environment. While their training could have influenced their behaviors, the apparent transferability of decision-making and reported realism of the system could extend beyond assessment to create new opportunities for firearm training and education. As the participants in the study made legally and situationally appropriate decisions, this also supports that training could influence safer behavior and decision-making. Whether as a complement to traditional training or a substitute for firing range practice in areas with limited access, VR systems could extend what had been almost exclusively used for military and law enforcement to the gun-owning public.

Participants reported that their behaviors and decision-making in the VR system were transferable to real life. This suggests that VR could provide gun owners with opportunities to test their skills and decision-making in a wide range of scenarios they might encounter, including situations in which firearm use might or might not be appropriate. Of particular interest was the participants’ decision not to intervene. Several participants reflected that their own use of a firearm could have escalated a situation—by compounding existing tensions or introducing a new deadly threat. Future research should explore the extent to which reasons for not intervening may be transferable to other critical encounters, such as events where power dynamics, political ideologies, or other expected social norms might differ (1, 10).

A 2015 national survey found that only 60% of gun-owning adults had ever completed any kind of formal firearm training, let alone immersive situations to assess decision-making (11, 12). In the context of no national training standard, no training requirement in more than half of US states as a result of permitless carry laws, and high rates of public concealed carrying, VR systems may provide an appealing, cost-effective and low-stakes opt-in learning environment. Moreover, VR may have the potential to improve training quality and prevent improper firearm use (13–15).

In addition to better understanding behaviors and decision-making, this study offered important insights for system improvements. For example, some mechanical improvements could be easily implemented to improve realism with the firearm used in the simulation. Adding pneumatics, increasing the weight, and improving the feel of the firearm are key adjustments to the system that would improve the participants’ experience. Additionally, incorporating ever advancing artificial intelligence to increase the interaction between the participants and the VR system would be an important technological advancement. Future research should also explore demographic changes to elements of the scenarios to assess their impact on decision-making such as the age, race, and sex of both the cashier and NPC involved in the robbery or shoplifting.

These findings should be considered in the context of some limitations. As this was a pilot study, we relied on a convenience sample and most of our participants were highly trained with firearms. We also restricted our sample to gun owners in Maryland given that we were piloting the system and wanted gun owners who were generally subject to the same laws for purchase and carry. This limits the generalizability of our findings to the broader population of firearm owners. However, the fact that these individuals were highly experienced and reported that they behaved the way they would in the real world intuitively supports that those with less training might also behave as they would in real life. Future research should examine the behaviors and decision-making of a more diverse pool of gun owners, both demographically and geographically, and those with varying levels of familiarity and experience with firearms.

As VR becomes increasingly available, its use as an innovative tool for assessment and training warrants future exploration. Based on the reflections of gun owners who used this system, VR appears to be feasible for scenario-based training and an effective tool for understanding gun owner decision-making.

## Data Availability

The datasets presented in this article are not readily available because we do not have permission to share data from the debriefing interviews. Requests to access the datasets should be directed to Cassandra Crifasi, crifasi@jhu.edu.
